# Navigating Environmental Perceptions: Exploring the Impact of Political Orientation and Climate Change Beliefs on the Evaluation of the Local Environment

**DOI:** 10.1007/s00267-025-02215-0

**Published:** 2025-06-28

**Authors:** Ruslan Gunko, Lauri Rapeli, Matias Scheinin, Jenny Wikström, Nina Tynkkynen

**Affiliations:** 1 Åbo Akademi University, Turku, Finland; 2 Bioeconomy Research Team, Novia University of Applied Sciences, Raseborg, Finland; 3 Water and Environmental Engineering, Turku University of Applied Sciences, Turku, Finland

**Keywords:** People-nature relationships, Environmental perceptions, Political preferences

## Abstract

Amid the global climate change crisis, the societal importance of the environment is becoming increasingly clear. Discussions on climate change and its impacts occur across various levels, yet the effects remain unclear for many. In this study, we examined the role of political orientation (left/right ideology) in shaping perceptions of climate change and local nature among residents in two municipalities in southwestern Finland. Our findings reveal a strong link between political orientation and changes in how local nature is perceived, particularly at the community level. While political opinions have a less significant influence on perceptions at the individual and national levels, they play a substantial role within communities. This suggests that political orientation becomes more influential in shaping environmental views as evaluations shift to the community level, emphasizing the interaction between political preferences and perceptions of local nature.

## Introduction

Perceptions of the natural environment are often defined as reflection of feelings about or awareness of the state of nature, and they shaped by personal values, emotions, and past experiences rather than technical expertise (Petrosillo et al. 2013; Hoyle et al. 2019). Individuals interpret and evaluate nature through subjective lenses, making sense of visual and sensory information based on what resonates with them personally (e.g., Schroeder 2002; Weber 2006). This interpretive process plays a key role in how people respond to environmental changes and risks. The accuracy of subjective evaluations made by citizens has been found to be accurate enough and may thus be considered for scientific or policy-making purposes (Chen et al. 2015; Gunko et al. 2022a).

It is important to remember that as with every subjective data, the perceptions of nature may be affected by a variety of factors such as previous experience (Steinwender et al. 2008), knowledge (Nahar et al. 2023), or emotional attachment to the particular area (Brown and Raymond 2007). Additionally, it has been found that the socio-economic status of individuals affects the way how they see the natural environment (Faeth et al. 2011; Jim and Shan 2013; Ellis et al. 2019). This is important to keep in mind due to the cumulative importance of socio-economic factors and the natural environment for people’s well-being (Durand 2015).

The perceptions are a relevant area of research: it was previously found that they play a key role when it comes to the effect of environmental factors on life quality and well-being of citizens (Gunko et al. 2022b). The role of nature for people’s well-being is continuously rising in the last decades and stimulates extensive research (Mayer et al. 2009; Richardson et al. 2016) and discussions in the media (Legagneux et al. 2018), not least because of the increased awareness of anthropogenic impacts on the environment (McGill et al. 2015; Newbold et al., 2015). A number of scholars have shown that the connection to nature and its adequate state are of vital importance for the life quality of an individual (White et al. 2013; Capaldi et al. 2015). Specifically, the positive link between nature and the improvement of health conditions (physical and mental) is emphasized (Ulrich et al. 1991; Lafortezza et al. 2009; Nisbet et al. 2010; Parra et al. 2010; Bratman et al., (2019)). The connection to nature seems to have a positive effect on stress impact reduction (Björk et al. 2008; Soga et al. 2021) and can buffer the negative impact on the well-being of global stresses such as the pandemic of COVID-19 (Gunko et al. 2022c). Consequently, paying attention to how people view nature and how they evaluate its state is vitally important for the achievement of high life quality standards.

### Environmental Topics and Political Orientations

As a consequence of the increased environmental awareness, questions and problems related to nature have in recent decades become increasingly important among ordinary people and policymakers (Pidgeon 2012; Bender et al. 2019; Lalot et al. 2022). Environmental matters are discussed not only on a national level worldwide but they have also become an important part of international politics because the problems reach across national boundaries (Leichenko and O’Brien 2008). Questions regarding the environment and climate change are integrated in political discussions and almost all political movements have composed their own approach to these questions (Purdy 2009; McCright et al. 2016). This is also the case in Finland (see e.g. Koskimaa et al. 2021), a country in Northern Europe, the object of this study.

Thus, the questions regarding the environment (specifically concerning climate change issues) are also among the matters that depend on political orientation. For example, there is evidence that leftists demand and expect more pro-environment actions from governments (Eurobarometer 2009). Also, people’s perceptions of the risks of climate change and climate change beliefs seem to depend on the political orientation (Hu et al. 2017; McCright 2011; Ziegler 2017; Gregersen et al. 2020). Thus, people with opposite political views may evaluate nature differently. Moreover, a recent study shows that people on the left and right perceive disagreement over climate policies differently: left-wing supporters tend to think that political divisions on climate issues are stronger than they actually are, while right-wing supporters tend to believe the divide is smaller than it really is (Leviston et al., 2024). At the same time, another study underscores that individuals with conservative ideologies tend to perceive greater threat from climate change-related reforms than from climate change itself (Stanley et al. 2024). Although the relationship between political orientation and climate change beliefs has been widely studied, there remains limited research specifically examining how these factors jointly influence environmental perceptions across different levels or contexts.

At the same time, the world, especially Western nations including Finland (Greven 2016; Rodrik (2021); Norocel et al. 2022), are witnessing a surge in right-wing populism. Politicians aligning with this movement frequently exploit the issue of climate change to appeal to climate skeptics and bolster their support base (Lockwood 2018; Yan et al. 2021). This trend may endanger many environmental initiatives at various levels (Schaller and Carius 2019), and contribute to growing climate change skepticism in society, especially in the context of weakening trust in environmental institutions and policies (Krange et al. 2021). Additionally, political orientation, combined with education level and perceived risk of climate change, appears to play a significant role in shaping landowners’ preferences for mitigation strategies (Vehola et al. 2022).

Traditionally, scholars roughly group all political movements into three categories by their political spectrum: center, right- and left wings. It has been noted that right-wing political movements are usually associated with climate change skepticism and lower attention to environmental questions (Lockwood 2018; Duijndam and van Beukering 2021), and in some cases have an effect of attraction already established climate change sceptics (e.g., Knollenborg and Sommer 2023). Left-wingers, in turn, demonstrate more pro-environmental behavior, have long history of relationship with pro-environmentalism in Western countries and approach and adopt the “green movements” often sharing their common values and views (Neumayer 2003; Carter 2013; Maccaferri 2022). Centrists politicians tend to have more neutral and more flexible views regarding the environment depending on the current political agenda (Carter 2006; Huber et al. 2021). It is important to note that the relationship between political orientations and environmental attitudes is less clear and direct in Central and Eastern European countries due to a combination of historical, political and socio-economic factors rooted in complex legacies of state socialism and a less ideologically structured political environment (Baranowski et al., 2024).

It is natural for political movements and parties to actively seek to influence the opinions of ordinary people about the main political topics regardless of people’s political preferences or orientation, which often remain strongly self-defined for many individuals (Slothuus and Bisgaard 2021). Most individuals define their political orientation based on values, traditions, knowledge, and socio-economic status (Beck and Jennings 1991; Kriesi 1999; Piurko et al. 2011; Lindholm and Rapeli 2023). However, a number of scholars highlight also the influence of the media as well as the importance of feelings in this process (Schwartz et al. 2010; Salmela, Von Scheve (2017); Peterson et al. 2019). Whilst individuals may change their preferences in parties or movements, for example in terms of vote choice in a specific election, they rarely change their fundamental political orientations but stay loyal to the trends of the selected wing regarding common topics about finance, migration, social equality, etc. (Dalton and McAllister 2015).

Despite the connection between a person’s political orientation and his/her environmental attitudes, there is, also, a gap in the understanding of how this connection fluctuates at different levels, such as country, local, and individual levels. Climate change beliefs and perceptions of the impact of climate change may differ significantly depending on the level where impacts are experienced (Osberghaus and Fugger 2022). For example, the perception of individual impact from climate change tends to be heightened among individuals who have experienced evidence-based changes in their familiar natural environment. This firsthand experience often leads to a greater level of consideration for the issue (Weber 2016).

In Finland, local policies and programs carry particular importance due to the central role municipalities play in providing public services and contributing to residents’ overall well-being (Loikkanen and Nivalainen 2011; Vakkala et al. 2021). Finnish local municipalities have autonomy in decision-making, steering municipality’s activities and finances, and managing the strategy through democratically elected local councils (Sahamies et al. 2023). Therefore, the significance of local processes at the community level holds potentially great value for local residents. At the same time, it is important to mention that parties usually have similar support on country and municipal level elections, suggesting that Finnish voters often choose the same party consistently across different types of elections (Sandberg 2022).

This paper scrutinizes how political orientation impacts people’s perceptions of the natural environment in Finland. Specifically, we aim to investigate if the perceptions change regarding the perceived impacts of climate change at different levels (country, local and individual), and what role the political orientation plays in these relationships. The exploration of the linkages within different levels may shed light on the linkages between political orientation, revealing whether these correlations vary in strength across different levels. Our primary contribution to the existing literature is to provide more evidence and to highlight the significance of personal experience and pre-existing political orientations as potential key drivers of climate-change attitudes. Finland provides a suitable context for such a study, because the effects of climate change are becoming increasingly tangible there through milder winters and more extreme weather fluctuations (Mikkonen et al. 2015; Kivinen et al. 2017). Additionally, Finland is a sparsely populated country with only a few major cities, meaning that people potentially live closer to nature. Furthermore, the Finnish party system includes politically significant parties throughout the left-center-right spectrum, which provides the necessary ideological variation for the study. Given the significance of local-level politics for environmental policy, the Finnish case encompasses those components, which make it possible to analyze climate change attitudes in relation to personal experience and the different policymaking levels. With these characteristics, Finland is a theoretically interesting case and a case representative of other countries with similar party systems and levels of political representation.

In our study, we hypothesize that political orientation has a significant effect on people’s environmental perceptions. Based on previous findings of scholars, we assume that political orientation is often linked to climate change beliefs, but its effects on subjective perceptions of nature vary significantly. Moreover, we predict a strong effect of political orientations on perceived impacts of climate change. We hypothesize that this effect is stronger on the individual level in comparison to the local and country-scale levels, due to the significant role of experienced personally impacts (evidence-based changes in their familiar natural environment).

## Research design

### The Case Municipalities: Naantali and Masku in South-west Finland

**Fig. 1:**
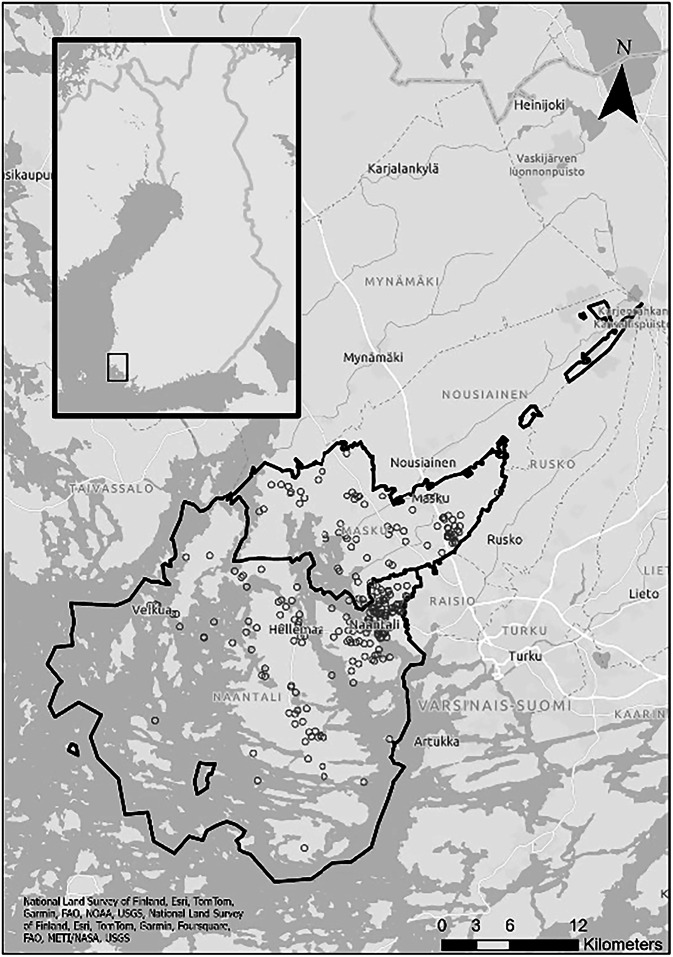
Study area with the points, where respondents to the questionnaire marked areas, they kept in mind during answering

The study area of our research consists of two neighboring municipalities Naantali and Masku located in the South-West part of Finland (Fig. 1). Both municipalities are in close distance to the large city Turku. Regardless of the fact that Naantali is almost twice as large by population (19,850 people in Naantali and 9642 in Masku), the population density in Masku is almost equal to Naantali (Official Statistics of Finland OSF (2022a)). These municipalities were chosen as case study municipalities because the inhabitants of both municipalities have access to different types of nature associated with coastal and forest environments. Moreover, these southwestern, coastal regions in Finland are among the first areas to experience the impacts of climate change. Additionally, we accounted the availability of high-resolution data about coastal water quality in these municipalities.

A further justification for the selection of the case municipalities is the similarity of political representativeness in both municipalities. Naantali and Masku inhabitants demonstrated similar voting preferences during the last elections on a country level (before the data collection) when most votes were given to the right-wing parties (see details in Table 1). It is important to note that National Coalition Party (NCP) is often grouped with right-wing parties, its environmental track record reflects more centrist tendencies. According to 2023 voting advice application data, NCP is more ecologically oriented than the Finns Party, though less so than left-wing green parties, suggesting a middle-ground position on climate and environmental policy (Isotalo, Rapeli (2024)).

**Table 1:** The Latest Election Results in the Study Area.

	Naantali	Masku
Municipal elections 2021	1. National Coalition Party 36,1% 2. The Finnish Social Democratic Party 19,7% 3. The Finns Party 16,3%	1. National Coalition Party 28,3% 2. The Finns Party 23,3% 3. The Finnish Social Democratic Party 15,5%
Parliamentary Elections 2023	1. National Coalition Party 35% 2. The Finns Party 21,7% 3. The Finnish Social Democratic Party 16,1%	1. The Finns Party 28,3% 2. National Coalition Party 24,7% 3. The Finnish Social Democratic Party 15,3%

The table highlights the parties that received the highest number of votes (Ministry of Justice, 2024)

## Data Collection

**Fig. 2:**
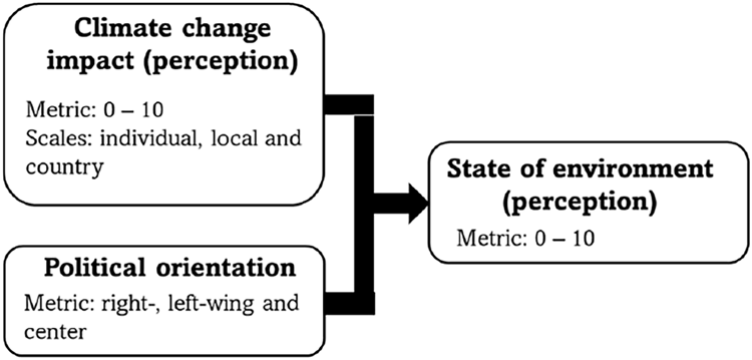
The research design of the study

We collected our data by surveying people about their perceptions of natural environments and climate change impact, and political preferences. The questionnaire was built in Survey 123 software and consists of a set of questions about the state of the environment, frequency of visits nature, well-being, respondent’s political orientation, party choice, and people’s socio-demographic status. All respondents were asked to point out the place about which they think when answering questions regarding the natural environment. The questionnaire was shared online and advertised by using Facebook advertisements targeting the adult population from 18 years old in both Naantali and Masku. Additionally, it was shared in the biggest local Facebook communities in the municipalities. To increase the share of participants, we used a motivation lottery with the draw of three supermarket gift cards. In this study, we used only people’s responses to questions regarding the subjective evaluation of the environment, evaluations of the perceived impact of climate change on individual, local, and country levels, and people’s political orientation (see details in Fig. 2). The collected data regarding other matters was used in other studies (the full questionnaire can be found in Appendix 1).

For answers to the questions about the subjective evaluation of the natural environment, we proposed to respondents to assess it on a 0–10 scale, where a higher score is associated with a better state of the environment. Similarly, the questions regarding the perceived climate change impact had a 0–10 assessment scale, where the higher score was associated with the stronger impact seen by the respondent. Our decision to use personally perceived climate change impact question is motivated by the idea that individual experiences with climate change influence personal beliefs about it (e.g., Osberghaus and Fugger, 2022). Finally, people had the possibility to share their political orientation by placing a mark on a 0–10 assessment scale, where “0” meant the extreme left political preference, while “10” – extreme right political orientation. Later, responses to the questions about political orientation were grouped into three categories based on the following logic: 0–4 were coded as left-wing political orientation, 5 – central, and 6–10 as right-wing political orientation. The decision to define only the value “5” as centrist is consistent with established practices in political science research employing left–right self-placement scales (e.g., Van Ditmars, 2023). It is widely recognized that individuals who do not identify with either end of the political spectrum tend to position themselves precisely in the middle of the scale. Moreover, there is broad agreement in the literature that even slightly off-centre responses (e.g., 4 or 6) typically indicate some degree of ideological leaning toward the left or right, respectively. This classification approach has been adopted in several studies aiming to clearly distinguish genuinely centrist respondents from those with mild ideological preferences (e.g., Aybar et al., 2024).

We corrected the collected survey data for age by applying a post-survey weight based on the population age structure in Naantali and Masku (Official Statistics of Finland OSF (2022b)) using the formula

$${\omega }_{i}=\frac{N{K}_{i}}{{n}_{i}},$$

where $N$ is the number of respondents, ${K}_{i}$ is desired distribution in the age group and ${n}_{i}$ is amount of respondents from the following age group. By this we corrected data and made it representative of population structure.

After the data was checked and we sorted out incomplete answers. In total, we got 270 responses that were suitable for further analysis (the distribution of political orientations is presented in Table 2). We used linear models to test whether the subjective evaluation of the state of natural environment can be predicted by the interaction between political orientation of the person and the climate change impact perceptions on different levels. For this we had three models presented in Table 3. The statistical analysis was done in R Studio (version 1.2.5042).

**Table 2:** The Distribution of Answers by Political Orientations

	Naantali	Masku	Total
**Left**	56	22	78
**Center**	31	14	45
**Right**	96	51	147

**Table 3:** List of Linear Models Used in the Analysis

*Model*	Response variable	Explanatory variables
1	Subjective evaluation of the environment	Climate change impact perception on individual level * Political orientation
2	Subjective evaluation of the environment	Climate change impact perception on local level * Political orientation
3	Subjective evaluation of the environment	Climate change impact perception on country level * Political orientation

## Results

### The Perceptions of Environment, Climate Change Impact and Political Orientation: Individual Level

**Fig. 3:**
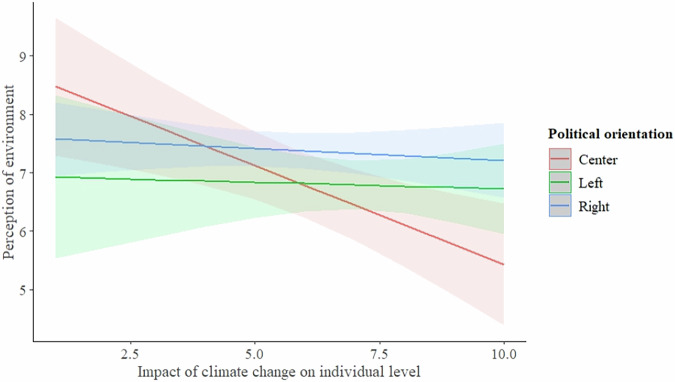
The interaction between climate change impact perception on individual level and political orientation of the respondent, and its effect on perception of natural environment

In our analysis, we tested the link between the subjective evaluation of the state of the natural environment and the political orientation of the person in connection to the perceived climate change impact on different levels (individual, local and country levels). First, we tested the potential connection on the individual level. We found that the way how the individual feels the impact of climate change significantly affected the evaluation of the state of the natural environment (F = 7.03; *p* = 0.008). At the same time, individual’s political orientation had no effect on the perception of nature (F = 2.99; *p* = 0.114). The interaction between individual-level perceptions of climate change impact and political orientation showed a weak association (F = 2.18; *p* = 0.052). Individuals who identified as right-wing tended to rate the environment more highly than those with other political orientations, particularly under conditions of greater perceived personal impact from climate change (Fig. 3; statistical details in Table 4).

**Table 4:** Linear Model of Relationship between Subjective Evaluation of Environment and Climate Change Impact Perception on Individual Level and Political Orientation of the Respondent

Dependent Variable	Independent Variables	Estimate ± SE	DF	F	*P*
Perception of environment	Intercept	**8.87** ± 0.71***			
	Climate change impact (individual level)	−**0.34** ± 0.11**	1	7.03	**0.008**
	Climate change impact (individual) by political orientation	Left 0.29 ± 0.16; **Right 0.31** ± 0.13*	2	2.18	**0.052**
	Political orientation	Left −1.78 ± 1.10; Right −1.27 ± 0.81	2	2.99	0.114

### The Perceptions of Environment, Climate Change Impact and Political Orientation: Local Level

**Fig. 4:**
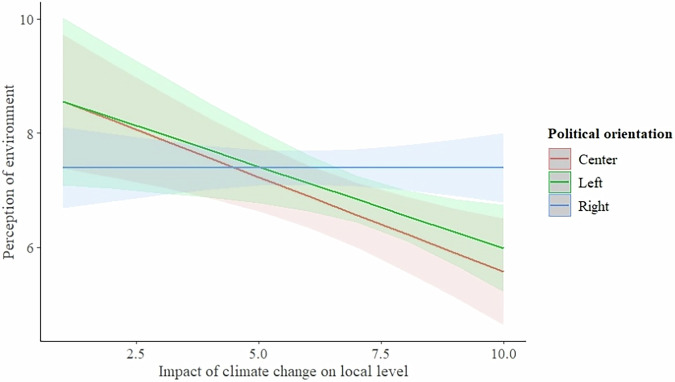
The interaction between climate change impact perception on local level and political orientation of the respondent, and its effect on perception of natural environment

Second, we examined the relationship between the subjective evaluation of the state of the natural environment and the political orientation of the person in connection to the perceived climate change impact on the level of municipality (local level). Our results showed that the climate change perception impact on the local level continues to have a significant effect on subjective evaluation of the environment (F = 10.28; *p* = 0.0015) and the political orientation effect was absent (F = 2.19; *p* = 0.114). However, the effect of interaction between these variables and the effect on perception of nature was significantly higher than on individual level. We found that on the local level right-wing-oriented respondents evaluated the environment significantly higher than others and this effect was more prominent (Fig. 4; statistical details in Table 5).

**Table 5:** Linear Model of Relationship Between Subjective Evaluation of Environment and Climate Change Impact Perception on Local Level and Political Orientation of the Respondent

Dependent Variable	Independent Variables	Estimate±SE	DF	F	*P*
Perception of environment	Intercept	**8.92** ± 0.69***			
	Climate change impact (local level)	**−0.33** ± 0.10**	1	10.28	**0.0015**
	Climate change impact (local) by political orientation	Left -0.01 ± 0.16; **Right 0.34** ± 0.12**	2	5.37	**0.0051**
	Political orientation	Left 0.25 ± 1.16; Right -1.56 ± 0.81	2	2.19	0.114

### The Perceptions Of Environment, Climate Change Impact And Political Orientation: Country Level

**Fig. 5:**
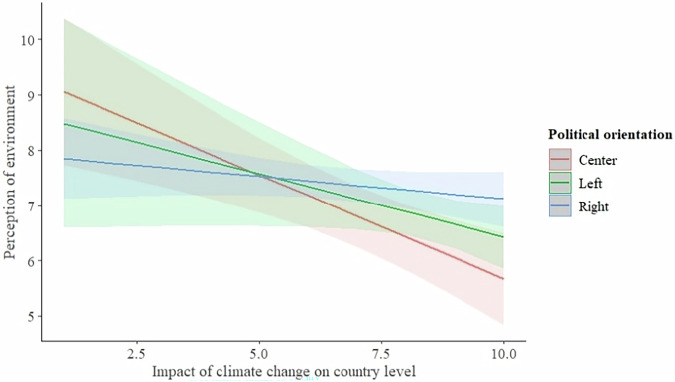
The interaction between climate change impact perception on country level and political orientation of the respondent, and its effect on perception of natural environment

Finally, we tested if the effect is changing when we consider respondents’ perceptions of climate change on the country level. We found a similar trend, when the climate change perception significantly affected people’s subjective nature evaluation (F = 16.93; *p* < 0.001), while the political orientation effect was absent (F = 1.52; *p* = 0.221). Again, the effect of interaction between climate change impact perception and political orientation on the country level was weak (F = 3.35; *p* = 0.036), but the observed previously trend that right-wing-oriented people rated the environment more favorably than left and center-oriented individuals, despite perceiving similarly high levels of climate change impact conditions (Fig. 5; statistical details in Table 6).

**Table 6:** Linear Model of Relationship between Subjective Evaluation of Environment and Climate Change Impact Perception on Country Level and Political Orientation of the Respondent

Dependent Variable	Independent Variables	Estimate ± SE	DF	F	*P*
**Perception of environment**	Intercept	**9.51** ± 0.78***			
	Climate change impact (country level)	**−0.38** ± 0.11***	1	16.93	**<0.001**
	Climate change impact (country) by political orientation	Left 0.12 ± 0.16; **Right 0.30** ± 0.12*	2	3.35	**0.036**
	Political orientation	Left -0.59 ± 1.34; Right -1.57 ± 0.89	2	1.52	0.221

The alternative analysis, which includes control variables such as age, gender, and education level, is presented in Appendix 2.

## Discussion and Conclusions

Our findings demonstrate that there is a strong link between the perception of climate change impact and perception of the natural environment on all studied levels from individual to the country level. Firstly, we found that people in general reflect their feelings about the impact of climate change in their evaluation of nature. In other words, when assessing the state of nature, people consider the impact of climate change as they perceive it on the individual, local and country levels. This aligns with previous findings noting that people rather accurately perceive the changes in their area and can link these with the state of the environment (Freihardt 2024). Moreover, it highlights that on the individual level, people directly associate the experienced changes in nature with the consequences of climate change (Reser and Bradley 2020).

Second, we tested the link between self-reported political orientation and subjective evaluation of the natural environment. The respondents expressed a high level of inclusion in the political life on the country and community levels. Almost 80% of people who participated in the survey reported interest in politics on the country level and more than 76% expressed interest in politics on the community level. Furthermore, almost 93% of the respondents said they voted in the last Parliamentary elections. Interestingly, regardless of the worldwide trend of the significant differences in evaluating nature among people who identify politically as left-wing or right-wing (Lind et al. 2023), we did not find any such connection in analyzing respondents’ responses from Naantali and Masku. This could be a reflection of the nature of the Finnish party system, where nearly all options regarding government coalitions are possible. Finland has a strong tradition of consensus-seeking, meaning that parties are generally reluctant to exclude others from cooperation. Consequently, parties from all ideological leanings are usually able to co-govern, which may result in less partisan division in certain policy areas, such as the climate, although there is also disagreement among the parties. However, the disagreement usually remains on a moderate level and local-level politics do not necessarily follow the agenda and dynamics of national-level party competition.

Finally, our results show that the perceived impact of climate change has a different association with environmental perceptions depending on political orientation. On all studied levels we found a similar trend indicating that people who identify themselves as right-wing voters evaluate the natural environment significantly differently (i.e., they perceive it more favorably) than people with center and left-wing political orientation. The effect is more pronounced on the local level, where right-wing politically oriented respondents tend to evaluate the environment positively regardless of the reported high climate change impact on the local level. The difference between right-wing oriented and other orientations regarding the perceived climate change impact can partly be explained by the political context of the studied municipalities (both governed by right-wing parties) and the corresponding distribution of our sample, which reflects a strong right-wing preference among respondents. Additionally, general climate change skepticism was found to be common among right-wing oriented parties and their voters (Lockwood 2018). This skepticism often involves doubts not only about the severity of climate change but also about its anthropogenic origins - a distinction that our study did not explicitly address, potentially influencing how participants interpreted and responded to questions about climate change impact. Additionally, this difference in views may be shaped by cultural worldviews related to fundamental beliefs about society and nature, as suggested by the Cultural Theory of Risk. As the theory indicates, these differing worldviews (right-wing vs. left-wing) can lead to varied interpretations of climate change impacts, potentially affecting how individuals evaluate the natural environment (McNeeley and Lazrus, 2014). It is important to note that our study did not ask participants to reflect on the origin of climate change: whether they believe it is caused by human activity (anthropogenic) or by natural processes. This omission could have a significant impact on how individuals interpret climate-related questions, as beliefs about the cause of climate change are closely tied to broader ideological and cultural worldviews. As a result, our findings may be limited by a reduced ability to differentiate between distinct climate change belief profiles.

The more pronounced link on the local, rather than other levels, found in our research highlights the significance of the changes taking place on the community level for local inhabitants, as they potentially are more tangible for inhabitants than country level changes. More to the point, municipalities in Finland have a significant role in decisions towards sustainable changes in infrastructure and land-use planning, and in some cases they even have monopolized the processes of sustainable transformations (Klein et al. 2016; Vaden et al. 2019; Salmi et al. 2022). For example, municipalities have significant control over urban planning processes, allowing them to respond to climate change impacts and guide construction activities toward more sustainable decisions and solutions (Tynkkynen et al. 2025). The strong role of municipalities in Finland (and elected municipal councils as representants of the community) may provoke differences in views between right-wing politically oriented respondents and others on a relationship between the perceived impacts of climate change and the perceptions of the state of the environment. The general approach to climate change policies in the programs of populist parties, which often prioritize socio-economic factors over environmental concerns (Vihma et al. 2020), may stimulate their voters to downgrade the importance of the environmental problems on a local level as well. The findings of this paper also have implications for our understanding of climate adaptation justice from the perspective of partisan ideology, and it would be wise to examine this further in a separate study.

As noted, an increasing interest in understanding the role of nature in people’s well-being has been evoked among scholars and policymakers over the last decade (Capaldi et al. 2014; Pritchard et al. 2020; Richardson et al. 2021). However, the role of nature seems to be more pronounced for life quality when looking at how individuals perceive the state of natural environments (Gunko et al. 2022b). The results of this paper highlight the key role of municipalities and their policy choices in people’s perceptions of nature and impacts of climate change. This is particularly important in contexts such as Finland, where municipalities traditionally play a significant role in the provision of human well-being due to their high autonomy in decision-making and service delivery.

## Supplementary information

Appendix 1

Appendix 2
