# Supplementary material for: Navigating Environmental Perceptions: Exploring the Impact of Political Orientation and Climate Change Beliefs on the Evaluation of the Local Environment
Source: Environ Manage. 2025 Jun 28;75(10):2647–58. doi: 10.1007/s00267-025-02215-0 (PMC12457493; doi:10.1007/s00267-025-02215-0)
Supplement: Supplementary file 1 — Appendix 1 [file 267_2025_2215_MOESM1_ESM.docx]

Thank you for agreeing to take part in this important survey measuring quality of life and state of the environment in Naantali and Masku. In this questionnaire we will ask questions regarding the state of environment in your municipality and your relation to that environment. This questionnaire only **takes 2-3 minutes** to complete. The study is part of the research conducted in the Sea research team at Åbo Akademi University and the results of the study will be used in future scientific publications. Your answers and all related information will be kept confidential.

By filling out the survey you can join the lottery and win 50€ S-Group gift card. Please, fill out the survey carefully, and if you wish to participate in the lottery, add your contact information.

On behalf of the research team,

Ruslan Gunko, Project Researcher

e-mail: ruslan.gunko@abo.fi

1. Are you… a) female b) male c) prefer not to say
2. What year were you born?
3. What is the highest level of education you have completed?
4. primary education; b) high school; c) vocational school or equivalent d) polytechnic university or equivalent e) university f) other
5. Which of the following comes closest to how you feel about your household's income nowadays?
6. living comfortably on present income b) coping on present income c) difficult on present income d) very difficult on present income
7. How is your health in general? Would you say it is…
8. very good b) good c) fair d) bad e) very bad
9. How you would assess the threat of climate change for humankind. Please choose a number from 0 to 10, where 0 means that you do not see any threat of climate change and 10 means that the threat is critical for our existence.
10. How you would assess the impact of climate change on the environmental conditions of your municipality. Please choose a number from 0 to 10, where 0 means that you do not see any impact of climate change and 10 means that the impact caused serious negative changes in environmental conditions.
11. How you would assess the impact of climate change on you personally. Please choose a number from 0 to 10, where 0 means that you do not feel any effect of climate change and 10 means that the effect is huge.
12. Place the pointer on the map approximately the place important for your everyday life in the municipality. This gives us an idea of which area in your municipality you are thinking about when answering our questions. You can place the pointer by clicking on the right place on the map. You can zoom using the plus and minus symbols.

In the next questions please think about the environment, which is close to the place you mentioned above.

In the next questions please think about the environment, which surrounds pointer you placed on the map in the question 9.

1. How would you assess the state of the natural environment in the area you pointed out as important for your everyday life? Please choose a number from 0 to 10, where 0 means it is very bad and 10 means it is excellent.
2. How close would you say you live to nature (e.g., green areas, natural forest, sea water)? Please choose a number from 0 to 10, where 0 means you do not live close to nature at all (e.g. in an apartment in the center of a town) and 10 means that you live surrounded by nature (e.g. on a farm or similar).
3. How often do you visit forest areas in close vicinity? a) never or almost never b) about once a month c) about once a week d) daily or almost daily e) cannot say
4. How rich do you think is the closest forest in terms of species of plants and animals? Please choose a number from 0 to 10, where 0 means it is poor and 10 means that it is rich.
5. To what extent does the permanent (related to land-use changes) or temporal (related to forest cultivation for example) affect how often you visit the closest forest? Please choose a number from 0 to 10, where 0 means “changes do not affect how often I visit the forest” and 10 means that the changes affect your forest visits.
6. How often do you visit nearby coastal areas by the sea? a) never or almost never b) about once a month c) about once a week d) daily or almost daily e) cannot say”
7. How would you assess the quality of the coastal waters environment in close vicinity to the area you pointed out as important for your everyday life? Please choose a number from 0 to 10, where 0 means it is very bad and 10 means it is excellent. If your home or cottage far away from any water body, give your estimation in the nearest water body to your house.
8. In your assessment, how important is the state of the environment in the area you pointed out as important for your everyday life for your overall wellbeing? Please choose a number from 0 to 10, where 0 means it does not matter at all and 10 means it is extremely important.
9. All things considered, how satisfied would you say you are with your life these days? Please tell me on a scale from 0 to 10, where 0 means very dissatisfied and 10 means very satisfied.

In the next questions we would like to ask questions related to your political preferences.

1. How often do you discuss politics with friends or family members? a) Never or almost never b) Occasionally c) Pretty often d) Every day or almost every day
2. How interested are in politics in general? a) not at all b) only a little c) somewhat d) very e) cannot say
3. How interested are you in local politics? a) not at all b) only a little c) somewhat d) very e) cannot say
4. In politics people sometimes talk of ‘left’ and ‘right’. Where would you place yourself on a scale from 0 to 10 where 0 means extreme left and 10 means extreme right?
5. A parliamentary election was recently held in Finland. Many people do not vote nowadays in elections. Did you vote in the recent parliamentary election? a) yes b) no c) do not want to say
6. If you responded in the previous question “yes”. What party did the candidate represent who you voted for?
7. Do you always vote for the same party in local and parliamentary elections? a) yes, always b) usually, but not always c) I always vote for different parties d) I do not vote regularly in both local and parliamentary elections
8. On a scale from 0 to 10, how important would you say it is for you that the party you vote for emphasizes environmental values? 0 means it is not at all important and 10 means it is decisively important for you.

Thank you!

Please, if you wish to participate in the lottery, leave your contact details (e-mail or phone number).
