# Supplementary material for: Navigating Environmental Perceptions: Exploring the Impact of Political Orientation and Climate Change Beliefs on the Evaluation of the Local Environment
Source: Environ Manage. 2025 Jun 28;75(10):2647–58. doi: 10.1007/s00267-025-02215-0 (PMC12457493; doi:10.1007/s00267-025-02215-0)
Supplement: Supplementary file 2 — Appendix 2 [file 267_2025_2215_MOESM2_ESM.docx]

**Appendix 2**

*Table 8. Linear model of relationship between subjective evaluation of environment and climate change impact perception on individual level and political orientation of the respondent (with age control variable included)*

| Dependent Variable | Independent Variables | Estimate ± SE | | DF | F | P |
| --- | --- | --- | --- | --- | --- | --- |
| Perception of environment | Intercept | **9.31 ± 0.81***** |  | |  |  |
|  | Climate change impact (individual level) | **-0.34 ± 0.11**** | 1 | | 6.55 | **0.011** |
|  | Climate change impact (individual) by political orientation | **Left 0.32 ± 0.16***;  **Right 0.31 ± 0.12*** | 2 | | 3.28 | **0.039** |
|  | Political orientation | Left -1.88 ± 1.07;  Right -1.28 ± 0.79 | 2 | | 2.15 | 0.118 |
|  | Age | -0.01 ± 0.01 | 1 | | 1.03 | 0.311 |

*Table 9. Linear model of relationship between subjective evaluation of environment and climate change impact perception on local level and political orientation of the respondent (with age control variable included)*

| Dependent Variable | Independent Variables | Estimate ± SE | | DF | F | P |
| --- | --- | --- | --- | --- | --- | --- |
| Perception of environment | Intercept | **9.45 ± 0.81***** |  | |  |  |
|  | Climate change impact (local level) | **-0.34 ± 0.11***** | 1 | | 9.91 | **0.0018** |
|  | Climate change impact (local) by political orientation | Left 0.06 ± 0.15;  **Right 0.35 ± 0.12**** | 2 | | 5.27 | **0.0056** |
|  | Political orientation | Left -0.17 ± 1.11;  **Right -1.64 ± 0.81*** | 2 | | 2.22 | 0.111 |
|  | Age | -0.01 ± 0.01 | 1 | | 0.82 | 0.364 |

*Table 10. Linear model of relationship between subjective evaluation of environment and climate change impact perception on country level and political orientation of the respondent (with age control variable included)*

| Dependent Variable | Independent Variables | Estimate ± SE | | DF | F | P |
| --- | --- | --- | --- | --- | --- | --- |
| Perception of environment | Intercept | **10.07 ± 0.89***** |  | |  |  |
|  | Climate change impact (country level) | **-0.39 ± 0.11***** | 1 | | 16.05 | **<.001** |
|  | Climate change impact (country) by political orientation | Left 0.17 ± 0.16;  Right 0.32 ± 0.12 | 2 | | 3.58 | **0.029** |
|  | Political orientation | Left -0.89 ± 1.32;  Right -1.66 ± 0.88 | 2 | | 1.55 | 0.213 |
|  | Age | -0.01 ± 0.01 | 1 | | 1.13 | 0.289 |

*Table 11. Linear model of relationship between subjective evaluation of environment and climate change impact perception on individual level and political orientation of the respondent (with gender control variable included)*

| Dependent Variable | Independent Variables | Estimate ± SE | | DF | F | P |
| --- | --- | --- | --- | --- | --- | --- |
| Perception of environment | Intercept | **9.12 ± 0.71***** |  | |  |  |
|  | Climate change impact (individual level) | **-0.36 ± 0.11**** | 1 | | 6.62 | **0.011** |
|  | Climate change impact (individual) by political orientation | **Left 0.34 ± 0.15***;  **Right 0.28 ± 0.12*** | 2 | | 3.12 | **0.045** |
|  | Political orientation | Left -2.04 ± 1.07;  Right -1.05 ± 0.79 | 2 | | 2.18 | 0.115 |
|  | Gender | **Male -0.48 ± 0.23*** | 1 | | 4.16 | **0.042** |

*Table 12. Linear model of relationship between subjective evaluation of environment and climate change impact perception on local level and political orientation of the respondent (with gender control variable included)*

| Dependent Variable | Independent Variables | Estimate ± SE | | DF | F | P |
| --- | --- | --- | --- | --- | --- | --- |
| Perception of environment | Intercept | **9.13 ± 0.71***** |  | |  |  |
|  | Climate change impact (local level) | **-0.34 ± 0.11***** | 1 | | 9.95 | **0.0017** |
|  | Climate change impact (local) by political orientation | Left 0.06 ± 0.15;  **Right 0.31 ± 0.12*** | 2 | | 3.99 | **0.019** |
|  | Political orientation | Left -0.18 ± 1.09;  Right -1.33 ± 0.81 | 2 | | 2.23 | 0.109 |
|  | Gender | **Ma**le -0.41 ± 0.23 | 1 | | 4.79 | **0.029** |

*Table 13. Linear model of relationship between subjective evaluation of environment and climate change impact perception on country level and political orientation of the respondent (with gender control variable included)*

| Dependent Variable | Independent Variables | Estimate ± SE | | DF | F | P |
| --- | --- | --- | --- | --- | --- | --- |
| Perception of environment | Intercept | **9.84 ± 0.78***** |  | |  |  |
|  | Climate change impact (country level) | **-0.41 ± 0.11***** | 1 | | 16.28 | **<.001** |
|  | Climate change impact (country) by political orientation | Left 0.18 ± 0.16;  **Right 0.28 ± 0.12*** | 2 | | 2.85 | 0.059 |
|  | Political orientation | Left -0.94 ± 1.31;  Right -1.36 ± 0.87 | 2 | | 1.57 | 0.209 |
|  | Gender | **Male -0.55 ± 0.23*** | 1 | | 6.51 | **0.011** |

*Table 14. Linear model of relationship between subjective evaluation of environment and climate change impact perception on individual level and political orientation of the respondent (with education level control variable included)*

| Dependent Variable | Independent Variables | Estimate ± SE | | DF | F | P |
| --- | --- | --- | --- | --- | --- | --- |
| Perception of environment | Intercept | **8.73 ± 0.79***** |  | |  |  |
|  | Climate change impact (individual level) | **-0.34 ± 0.11**** | 1 | | 6.46 | **0.012** |
|  | Climate change impact (individual) by political orientation | **Left 0.31 ± 0.16***;  **Right 0.30 ± 0.12*** | 2 | | 3.05 | **0.049** |
|  | Political orientation | Left -1.83 ± 1.08;  Right -1.17 ± 0.80 | 2 | | 2.29 | 0.103 |
|  | Education level | High education 0.05 ± 0.23 | 1 | | 0.11 | 0.758 |

*Table 15. Linear model of relationship between subjective evaluation of environment and climate change impact perception on local level and political orientation of the respondent (with education level control variable included)*

| Dependent Variable | Independent Variables | Estimate ± SE | | DF | F | P |
| --- | --- | --- | --- | --- | --- | --- |
| Perception of environment | Intercept | **8.86 ± 0.79***** |  | |  |  |
|  | Climate change impact (local level) | **-0.34 ± 0.10**** | 1 | | 9.61 | **0.0021** |
|  | Climate change impact (local) by political orientation | Left 0.05 ± 0.15;  **Right 0.34 ± 0.12**** | 2 | | 4.94 | **0.007** |
|  | Political orientation | Left -0.05 ± 1.11;  Right -1.51 ± 0.81 | 2 | | 2.37 | 0.095 |
|  | Education level | High education 0.02 ± 0.23 | 1 | | 0.05 | 0.829 |

*Table 16. Linear model of relationship between subjective evaluation of environment and climate change impact perception on country level and political orientation of the respondent (with education level control variable included)*

| Dependent Variable | Independent Variables | Estimate ± SE | | DF | F | P |
| --- | --- | --- | --- | --- | --- | --- |
| Perception of environment | Intercept | **9.23 ± 0.84***** |  | |  |  |
|  | Climate change impact (country level) | **-0.39 ± 0.11***** | 1 | | 15.72 | **<.001** |
|  | Climate change impact (country) by political orientation | Left 0.15 ± 0.16;  **Right 0.31 ± 0.12*** | 2 | | 3.28 | **0.038** |
|  | Political orientation | Left -0.68 ± 1.33;  Right -1.51 ± 0.88 | 2 | | 1.69 | 0.185 |
|  | Education level | High education 0.16 ± 0.23 | 1 | | 0.49 | 0.484 |
